# Significance of the Patella Tendon-Reflex

**Published:** 1887-01

**Authors:** 


SIGNIFICANCE OF THE PATELLA TENDON-REFLEX.

It would seem that the countless observations and numerous articles and monographs that have appeared on this subject within the last ten years would have settled the significance of this symptom beyond doubt. But such does not seem to have been the case, as the recent discussion of the subject before the Chicago Medical Society shows.

Perhaps too much importance has been attached to the mere presence or absence of the knee-jerk. There are well-authenticated cases in which it is absent in healthy persons. Again it frequently persists in cases of undoubted ataxia, which is the disease most frequently associated with its loss.

The proportion of healthy people who have an absence of the reflex appears to be small. The wide difference of views on this subject is due, largely, to different modes of testing. Any testimony regarding the absence of the phenomenon, in which the tendon was struck when covered by the clothing, is probably of little value, or that in which the blow was given with the ulnar side of the hand or the tips of the fingers. In all cases in which the reaction is uncertain the knee should be bared and the tendon struck repeatedly over its entire surface. If there is still doubt, the patient should be directed to clasp the fingers and excite a vigorous voluntary contraction just as the tendon is struck. This method greatly lessens the inhibitory power of the brain by concentrating it on the upper segments of the cord. Since this suggestion of Jendrassik, the writer has never seen a healthy person in whom it was absent. This however does not agree with the experience of some others, particularly Professor Lester Curtis. Ross ("Diseases of the Nervous System," vol. I, p. 163) states that it is probable that the reaction may be absent in healthy persons, and quotes Berger, who examined 1,409 healthy individuals and found the reflex absent in 22, or 1.56 per centum.

It is conceded that Berger is a competent observer, and it was supposed that he knew how to elicit this reaction, but it is to be remembered that at the time his observations were made (February, 1879) the method of Jendrassik had not been pointed out. It is more than probable that, in the light of our present knowledge regarding the conditions under which this examination should be made, the percentage of Berger would have been very much lessened. Another circumstance of importance in this connection is the variability of this symptom in health and disease. In the same individual it will be at times very easy and at other times very difficult to elicit. Ross also mentions this fact (ibid p. 163). Another fact pointed out by Professor Curtis is that the views of those especially interested in neurology are based upon observations of dispensary and hospital patients, with weak nerves and still weaker bodies. He thinks that an examination of healthy, strong-willed and vigorous individuals would reveal a much larger proportion of those in whom the symptom is absent.

While the testimony is somewhat conflicting, we are justified in concluding that the *absence* of the symptom is a suspicious circumstance, yet, if other signs fail, it is not indicative of disease. The *loss* (i. e., those in whom it was present, but has disappeared) is a circumstance of the greatest moment, and almost certainly indicates grave disease of the central nervous system.

The presence of the reflex in certain cases of well-marked ataxia which have gone on to a fatal termination, should lead to caution in diagnosis as well as prognosis in those cases in which other symptoms of ataxia are present.

